# The Impacts of Spirituality and Religious Participation on the Emotional Well-Being of Widowed Older Adults in Southeast Nigeria

**DOI:** 10.1093/geroni/igad128

**Published:** 2023-11-15

**Authors:** Chidinma P Ukeachusim, Christopher O Okwor, Ekenedirichukwu Eze, Anuli B Okoli, Collins I Ugwu, Samuel O Ebimgbo

**Affiliations:** Department of Religion and Cultural Studies, University of Nigeria, Nsukka, Nigeria; Department of Abrahamic Religions, University of Religions and Denominations, Qom, Iran; Department of Religion and Cultural Studies, University of Nigeria, Nsukka, Nigeria; Department of Religion and Cultural Studies, University of Nigeria, Nsukka, Nigeria; Department of Religion and Cultural Studies, University of Nigeria, Nsukka, Nigeria; Department of Religion and Cultural Studies, University of Nigeria, Nsukka, Nigeria; Department of Social Work, University of Nigeria, Nsukka, Nigeria; Faculty of Catholic Theology, Julius-Maximilians University, Würzburg, Germany

**Keywords:** Emotional well-being, Religious activities, Southeast Nigeria, Spirituality, Widowed older adults

## Abstract

**Background and Objectives:**

The population of older individuals in Africa is increasing, and at a rapid rate. Although the numbers of these individuals increase, many African countries, including Nigeria, are devoid of state-sponsored welfare systems that address their well-being. This situation has placed the bulk of support on the family members including spouses. Studies have shown that spouses are considered to be veritable sources of support in later life. However, spousal death tends to reduce the perceived support including emotional support, which usually occasions lower well-being, life dissatisfaction, and higher mortality rates. This study ascertains the impact of spirituality/religious participation on the emotional well-being of widowed older adults.

**Research Design and Methods:**

The study was conducted in Enugu State, southeast Nigeria. The study implemented a qualitative approach to collect data from 71 widowed older adults, aged 60 and older. The thematic analytical method was used to analyze the generated data.

**Results:**

Participating in spiritual/religious activities like prayers, church programs/activities reading, studying, and meditating on the word of God have enabled the widowed older adults to buffer some of the emotional challenges.

**Discussion and Implications:**

Older adults should be encouraged to participate in spiritual and religious activities. Religious leaders should also make every effort to organize activities that will address the emotional needs of these individuals.

**Translational Significance:** Religious participation has been a crucial factor for adapting to certain life-threatening situations. Nigeria is experiencing an increase in the number of older adults; meanwhile, state-sponsored support programs for the well-being of these individuals seem to be minimal. Thus, informal support networks including spouses become important. However, spousal deaths tend to deplete available support including emotional support. This study ascertained the impact of spirituality/religious participation on the emotional well-being of widowed older adults. Involvement in religious/spiritual activities buffers the emotional challenges experienced by these individuals. Special programs to support these individuals by the religious leaders are encouraged.

Across the globe, there is a noticeable surge in the human population of those in the age range of 60 or older. In 2017, more than 900 million older adults lived across the globe, and this number may likely rise to more than 2 billion by 2050 (United Nations Department of Economic and Social Affairs, Population Division [UN DESA], 2017). Improved nutrition, healthcare facilities, education attainment, and healthy economic condition, which occasioned longevity, have led to an increase in the aging population (Tanyi et al., 2018). The experiences of longevity and increase in population aging have also led to the increase in the number of widowed older adults (Sullivan & Fenelon, 2014). Widowed older adults were projected to be about 350 million in 2020, and a greater percentage was likely to be female (Sasson & Umberson, 2014). China was found to have the greatest number of widowed older adults across the globe (National Bureau of Statistics [NBS], 2020; Harma, 2015). The report of the Federal Interagency Forum on Aging-Related Statistics (2016) revealed that about 34% of women and 12% of men who are older adults in the United States were widowed; meanwhile, the number increases as they grow older. In Africa, the exact number of widowed older adults is not readily available in literature, but van de Walle (2016) estimated that out of 10 African women, one is widowed.

Noticeable too is the apparent increase in the population of older individuals in Nigeria. By 2050, Nigerian older adults will constitute about 10% of the total population of the country, given that the total population of older adults is projected to increase to more than 25 million by 2050 (United Nations, Department of Economic and Social Affairs, 2017). This implies that the number of Nigerian widowed older adults may likely increase as the number of older adults increases. Similar to some other African countries, Nigeria has no documented data depicting the number of widowed older adults, but the study by Milazzo and van de Walle (2018) shows that the number of widows surpasses that of widowers. The authors noted that while only 11% of male older adults are widowers, 77% of female older adults are widows.

Obviously, the number of Nigerian older adults including the widowed is increasing when the nation is facing numerous challenges like poverty and epidemic (Adebanjoko & Ugwuoke, 2014), weakened traditional values, and lack of government-sponsored welfare programs (Ebimgbo, Nnama-Okechukwu, et al., 2022). Out of 133 million Nigerians that are regarded as multidimensionally poor (NBS, 2022) and roughly 71 million that live in extreme poverty (World Data Lab, 2023), older adults are predominant (Ebimgbo, Atama, et al., 2022). The poor economic state of Nigerian older adults occasioned by the lack of government-initiated intervention programs pushes these individuals to toil irrespective of their old age (Adisa, 2019). Nigeria had in the past demonstrated negative attitudes toward approving and implementing several state-sponsored bills for the well-being of older adults (Abayomi & Adetayo, 2019; Agbakwuru, 2018; Aiyede et al., 2015; Okoye 2020; PM News, 2018). The aging policy that was approved in 2021 by the Nigerian government, which appeared to bring much-anticipated succor to the plight of older adults (Lamai, 2021; News Agency of Nigeria, 2021), seems to be waning, due to failure of implementation after 2 years. Given the limited scope of state-sponsored support for Nigerian older adults, the informal support networks including family members become important sources of financial, health, health information, and emotional care to these older adults (Ebimgbo & Okoye, 2017). However, with a weakened condition of traditional values, the care from the informal support networks seems to be threatened (Ebimgbo, Nnama-Okechukwu, et al., 2022). These conditions have greatly scuffled the perceived support for these individuals and thereby negated their subjective well-being.

The roles of spouses in maintaining the well-being [health, material, financial, and emotional] of older adults in Nigeria cannot be overemphasized (Ebimgbo, Agwu, et al., 2021; Ebimgbo, Atama, et al., 2022; Ebimgbo, Chukwu, et al., 2021). Studies abound to show that the changes in family dynamics resulting from spousal deaths occasion poor support and well-being of the left-behind spouses (Holt-Lunstad, 2017; Isherwood, 2017; Sasson & Umberson, 2014; Williams et al., 2012). In most cases, spousal death culminates in several negative experiences like maltreatment, discrimination, and stigmatization from members of society (Onyima, 2022). Frustration and sadness are some of the challenging experiences of these individuals upon inadequate well-being (Ebimgbo, Nwachukwu, et al., 2022). Inadequate well-being of these individuals occasions other emotional and physical health problems including depression, maltreatment, and body pains, which usually lead to their death (Caetano et al., 2013; Melchiorre et al., 2013).

In a longitudinal analysis by Pena-Longobardo et al. (2021), individuals who are widowed experience poor well-being and worsening mental health. In the same vein, Kristiansen et al. (2019a, 2019b) revealed that widows develop mental disorders such as anxiety and depressive disorder consequent upon the deaths of their spouses, whereas widowers experience anxiety and depressive disorders. The experience of depressive disorders is prevalent in between 17% and 20% of widowed individuals (Kristiansen et al., 2019b). Among the Chinese, spousal deaths occasioned depression (Bi et al., 2022; Tian & Chen, 2022) and anxiety (Bi et al., 2022). Spousal deaths result in increased stress among Indians due to poor emotional and financial support (Ansari et al., 2023; Srivastava et al., 2021). In the United States, bereavement, especially that of older spouses, leads to severe depressive symptoms (Domingue et al., 2021), whereas Germans and Koreans experience anxiety, grief, depression, emotional loneliness, and social loneliness (Förster et al., 2019; Kim & Lyu, 2018). This is also evidenced in the systematic review and meta-analysis by Singham et al. (2021) and Szabó et al. (2020). Several authors have proven that depressive experiences due to spousal loss culminate in sadness or grief, poor judgment, sleeping problems, life dissatisfaction, guilt, and suicidal ideation (Battle, 2013; Brouwer et al., 2022).

According to Ebimgbo, Atama, et al. (2022), the well-being of older individuals including the widowed should be the primary focus of several support systems to enable them to achieve healthy aging. In Nigeria, the roles of various support systems like family, community, and friends in the care of older adults have been established (Ebimgbo, Agwu, et al., 2021; Ebimgbo, Atama, et al., 2022; Ebimgbo, Chukwu, et al., 2021; Ebimgbo, Nnama-Okechukwu, et al., 2022; Ebimgbo & Okoye, 2017; Mobolaji & Akinyemi, 2022). Other studies have also acknowledged the role of religious organizations in the care of older adults. Nigeria is divided into numerous religious groups including Christianity, Islam, and traditional/Oriental (National Open University of Nigeria, 2008). However, Wusu (2011) limited the major religions in Nigeria to two, Christianity and Islam, with Christianity having diverse denominations. Ebimgbo et al. (2018) found that church-based organizations are vital in the care of older adults in south-east Nigeria. Also, Ede et al. (2023) revealed that religious and spiritual participants are useful to the psychological health and the general well-being of older people in Nigeria. Among the South-western Nigeria population, the study by Mobolaji and Akinyemi (2022) discovered the significant role of religious organizations in the support of older adults, whereas in Bauchi, North-Eastern Nigeria, Yakubu and Namadi (2018) found that older adults who are Moslems deeply practice the activities of *Jeedo* to enable them to age in place and to achieve life satisfaction. Among the widows, studies have documented the roles of church-based organizations in reducing their challenges through financial, material, and information support (Agubuzu-Oyi, 2014; Zhiya, 2015).

The current study observed that these studies were unable to capture the relationship between religious and spiritual activities and emotional well-being of widowed older adults. The studies by Agubuzu-Oyi (2014) and Zhiya (2015) considered the views of widows without considering the widowers, a lacuna that the current study considered vital to fill. Thus, our study attempted to ascertain the impacts of religious and spiritual activities on the emotional well-being of widowed older adults. In this study, we acknowledged spirituality to be the pursuit for the sacred being by the widowed older adults, which in most cases enables them to achieve a profound relationship with the sacred, whereas religion entails gathering of widowed older adults for a common set of practices and beliefs, which is usually supported by routine ceremonies that provide them satisfaction with life.

The Conservation of Resources theory (COR), developed by Hobfoll (1989), was used to structure the study’s framework. The theory posits that in one’s struggle to retain, protect, and build resources, there are still some externalities that may threaten the loss of these treasured resources. Hobfoll avers that the loss of the resources culminates in the experience of emotional or psychological breakdown, though this loss might likely cause the individuals to reinvest in more resources. The resources may be in material form such as money and housing; it could be social, which includes social support and status, and could be psychological like personal mastery, and sense of autonomy (Hobfoll, 1989). Thus, spouses are seen as crucial sources of emotional support, which is an important resource in maintaining well-being, especially in old age. However, the loss of any of the spouses due to death usually culminates in the loss of these resources and thereby occasions the experience of emotional difficulties.

Conservation of resources theory also highlights the importance of resource regain, which to a great extent acts as an emotional buffer especially during loss of resources (Farkash et al., 2022). In the view of Wells et al. (1999), whenever individuals lose resources, they make every effort to regain them to enable them to secure their emotional strength for achieving desired goals. In most cases, when older adults lose any of their spouses, they resort to religious and spiritual activities, which are potential tools to regain the lost resources. In Africa, and Nigeria in particular, the lives of the people are well grounded in religion. Religion has permeated so much into the fabrics of people’s lives that Mbiti (1976, p. 1) describes Africans as “being so notoriously religious.” Nigerian older adults have been found to be hugely involved in the activities of spirituality and religion (Sampson, 2014; Zimmer et al., 2016). This is attributed to the notion that spirituality and religion are veritable tools for successful aging (Porte et al., 2017). The widowed older adults usually turn to spiritual and religious activities to buffer some of the challenges including anger occasioned by death of spouses (Carr, 2020). The participation of widowed older adults in religious and spiritual activities gives them hope and reasons to stay put and cope with the spousal loss (Neimeyer, 2005). Also, the belief of widowed older adults in God through spiritual activities provides them with needed attributes to overcome widowhood experiences including feelings of anger and injustice, and other emotional consequences (Davis et al., 2000). Pilger et al. (2017) revealed that spiritual activities are essential for the well-being of older adults, providing them with necessary support for life satisfaction and sense of purpose.

## Materials and Methods

### Designs, Setting, and Sampling

The descriptive-phenomenological study design was adopted as the design for the study. The design was adopted to enable the lived experiences of a phenomenon (Lopez & Willis, 2004; Willis et al., 2016). The design helped to describe the emotional challenges experienced by widowed older adults as well as the roles of religious or spiritual activities in ameliorating these challenges (Sauro, 2015). The study was conducted in the southeast geopolitical zone of Nigeria. Five states namely, Abia, Anambra, Ebonyi, Enugu, and Imo, make up the southeast part of Nigeria. Despite having other religious sects like African Traditional Religion and Islam, the southeast region is densely populated by Christians (NBS, 2012), predominantly dominated by Igbo ethnic groups (Pew Forum on Religion & Public Life, 2010). In southeast Nigeria, women are usually subjected to some harmful widowhood practices such as oath-taking at deities, drinking of water used in cleansing the dead spouses, being confined in a room with the deceased for hours, and chewing kolanut placed on the dead (Mezieobi et al., 2011). These practices often take a toll on the emotional well-being of these individuals.

The participants for this study were 71 (63 female and 8 male) widowed older adults aged 60 and older. The participants were recruited on the premise of age (60 years infinity), must be widowed, and available and willing to give their views on the topic under study. Through purposive, snowballing, and availability sampling, the participants for the study were selected. Out of the five states in southeast Nigeria, the researchers purposively selected Enugu State for the study. With a total population of 224,906 older adults, comprising 123,515 men and 101,391 women (National Population Commission, 2010), Enugu is among the states with a high number of older adults in the southeast. Thus, it is believed that the number of widowed older adults will be high as there is no official record regarding this in southeast Nigeria. Secondly, Enugu state is known for religious activities with different religious sects existing in the state; this allowed the researchers to obtain information from various individuals from divergent religious sects.

The researchers purposively selected two Local Government Areas (LGAs) out of 17 LGAs in Enugu State. The selected LGAs are Nsukka and Igbo-Eze North. The challenging experiences of widowers are rarely found in literature, especially in southeast Nigeria, so this study opted to capture the views of the widowers. A community was selected in each of the LGAs. At Nsukka LGA, Obukpa community was selected, whereas Uda was selected in Igbo-Eze North LGA. We purposively selected older adults who are widowed for the study because we believed that at this stage of life, they may not be interested in remarrying. Also, some of them may have been widowed for a long period, thereby giving them the edge to have experienced emotional challenges more than the younger ones. With the help of community leaders, we were able to identify some of the widowed older adults, and these identified older adults linked us to others who were available. In all, 38 participants (33 female and 5 male) were selected from Nsukka LGA, whereas 33 participants (30 female and 3 male) were selected from Igbo-Eze North LGA. During the recruitment, the date and time for the study were discussed as well as the information they needed to know regarding the study’s aims, anticipated risks, and benefits. The participants were assured of confidentiality and anonymity; hence pseudonyms are used in presenting the study findings instead of the real names of the participants. They were also given the privilege of withdrawing from participating in the study at any time.

The study methodology and the instrument were subjected to the approval of the faculty research committee, while the informed consent to participate in the study as well as permission to use a digital recorder were obtained verbally from the participants prior to their recruitment and discussions. We approached 81 widowed older adults during recruitment and 10 of them declined to participate in the study. Some of them were unwilling to participate, whereas others felt that the timing of the study was not convenient. To ensure the reliability or trustworthiness of the study data, we did a pilot study using widowed older adults in Nsukka LGA who were not among the participants selected for the main study. This enabled us to identify likely problems that would have emanated in the course of the study.

### Data Collection and Analysis

A qualitative approach using focus group discussions (FGDs) and in-depth interviews (IDIs) was adopted to collect data from participants. Six FGDs were conducted in the selected LGAs. Three FGDs were conducted in both Nsukka LGA and Igbo-Eze North LGA. The FGDs were all-female groups with 10 participants in each session. On the other hand, 11 IDIs were conducted with widowed older adults. Although eight IDIs were conducted with male participants, three IDIs were conducted with female participants. At Nsukka LGA, eight IDIs with five men and three women were conducted, whereas three IDIs with men were conducted at Igbo-Eze North LGA. The FGD guide and IDI schedule served as the instruments for data collection. The discussion guide and interview were semistructured to enable the researchers to probe further regarding their views on the topic under discussion. The discussion guide was designed to last between 40 and 55 min, whereas the interview guide was prepared to last between 20 and 25 min. Except for one male participant in IDI whose interview took place in his office, all other IDIs were conducted in the homes of the participants. On the other hand, the discussions with participants in Nsukka LGA were carried out in the village hall, whereas the discussion with participants at Igbo-Eze North LGA took place in primary school classrooms.

The inductive thematic analytical method was employed in the analyses of the transcripts and field notes (Braun & Clarke, 2006). The researchers did a verbatim transcription of the audio-taped data collected from the participants in the *Igbo* language. This was later translated into English with attempts to ensure the same meaning. After the translation, we devoted time to read the transcripts over time and in separate timings to identify and generate common and recurrent themes and subthemes. These common and recurrent themes and subthemes that were identified by the researchers were used to report the findings drawn from this study (Table 1).

**Table 1.** Guide of Questions for FGD and IDI, Emerged Themes, and Subthemes

S/N	Key Question	Themes	Subthemes
1	What are the emotional challenges experienced by widowed older adults in the community?	Loneliness	• No one to talk to • Creates vacuum
		Life dissatisfaction	• Unhappy at point of family progress • Better to die
		Anxiety/uneasiness	• Excessive thinking • Worry about financial responsibility
		Sadness/grief	• Receipt of inhuman treatment • Accusation from people
		Insomnia	• Much thinking about family well-being
2	What are the spiritual/religious activities widowed older adults are participating in?	Church activities	• Sunday services • Night vigils/prayer meetings • Morning mass
		Personal and family spiritual activities	• Bible reading/study • Meditating • Morning/night devotion
3	To what extent do spiritual and religious activities aid widowed older adults?	Aided to buffer the emotional challenges	• Encouragement • Comfort • Happy • Relief

*Notes*: FGD = focus group discussion; IDI = in-depth interview.

*Source*: Researchers’ fieldwork 2021.

## Findings

The characterizations of the background of the study are summarized in Tables 2 and 3. The data in the tables show that women (*n* = 63) participated in the study more than men (*n* = 8). This indicates that women were more available during the study. The finding may not be out of place, because studies have shown that many men who lost their spouses tend to remarry within a short period of time (Makena, 2020), while for a myriad of reasons, a greater number of women do not wish to remarry in this part of the world (Smith et al., 1991). Only 15 participants noted that their periods of widowhood are less than 5 years.

**Table 2.** Sociodemographic Characteristics of IDI and FGD Participants in Nsukka Local Government Areas

S/N	Pseudonym	Sex	Type of Study	Age	Religion	Education Status	Occupation	Monthly Income	Health Status	Period of Widowhood
1	Mr. Onu	M	IDI	67	Christianity	SSCE	Trader	>₦30,000	Healthy	6 months
2	Mr. Uke	M	IDI	70	Muslim	SSCE	Farmer	<₦30,000	Unhealthy	3 months
3	Mr. Ikwu	M	IDI	65	Christianity	Tertiary	Teacher	<₦30,000	Healthy	6 months
4	Mr. Onye	M	IDI	75	Christianity	Secondary	Trader	<₦30,000	Healthy	2 years
5	Prof. Kodo	M	IDI	65	Christianity	Tertiary	Lecturer	>₦30,000	Healthy	16 years
6	Mrs. Nky	F	IDI	60	Christianity	Primary	Trader	>₦30,000	Unhealthy	13 years
7	Mrs. Ros	F	IDI	64	Christianity	No education	Trader	>₦30,000	Healthy	11 years
8	Mrs. Flo	F	IDI	N/I	Christianity	FSLC	Farming	<₦30,000	Unhealthy	22 years
9	Mrs. Ono	F	FGD	60	Christianity	No education	Farming	<₦30,000	Healthy	4 years
10	Mrs. Ife	F	FGD	N/I	Christianity	No education	Unemployed	<₦30,000	Unhealthy	20 years
11	Mrs. Mod	F	FGD	96	Christianity	No education	Unemployed	<₦30,000	Unhealthy	30 years
12.	Mrs. Ido	F	FGD	N/I	Christianity	No education	Farming	<₦30,000	Healthy	N/I
13	Mrs. Oki	F	FGD	N/I	Christianity	No education	Unemployed	<₦30,000	Unhealthy	20 years
14	Mrs. Ibo	F	FGD	N/I	Christianity	No education	Farming	<₦30,000	Unhealthy	30 years
15	Mrs. Obu	F	FGD	60	Christianity	Primary	Trader	<₦30,000	Healthy	15 years
16	Mrs. Ubo	F	FGD	N/I	Christianity	No education	Unemployed	<₦30,000	Healthy	N/I
17	Mrs. Uge	F	FGD	68	Christianity	No education	Trader	<₦30,000	Unhealthy	13 years
18	Mrs. Okwu	F	FGD	N/I	Christianity	No education	Trader	<₦30,000	Unhealthy	19 years
19	Mrs. Som	F	FGD	N/I	Christianity	No education	Trader	<₦30,000	Unhealthy	20 years
20	Mrs. Ekwi	F	FGD	N/I	Christianity	No education	Farmer	<₦30,000	Unhealthy	N/I
21	Mrs. Ikwe	F	FGD	N/I	Christianity	No education	Farmer	<₦30,000	Healthy	N/I
22	Mrs. Mar	F	FGD	N/I	Christianity	No education	Farming	<₦30,000	Healthy	26 years
23	Mrs. Jo	F	FGD	60	Christianity	No education	Farming	<₦30,000	Unhealthy	25 years
24	Mrs. Sob	F	FGD	N/I	Christianity	No education	Farming	<₦30,000	Healthy	N/I
25	Mrs. Bose	F	FGD	60	Christianity	BSc	Civil servant	>₦30,000	Healthy	31 years
26	Mrs. Ose	F	FGD	70	Christianity	No education	Trading	<₦30,000	Healthy	30 years
27	Mrs. Idu	F	FGD	85	Christianity	No education	Unemployed	<₦30,000	Unhealthy	5 years
28	Mrs. Aja	F	FGD	67	Christianity	Primary	Trading	<₦30,000	Healthy	15 years
29	Mrs. Cok	F	FGD	85	Christianity	No education	Trading	<₦30,000	Unhealthy	17 years
30	Mrs. Fana	F	FGD	65	Christianity	Primary	Trading	<₦30,000	Unhealthy	10 years
31	Mrs. Ana	F	FGD	60	Christianity	Secondary	Trading	<₦30,000	Healthy	8 years
32	Mrs. Mab	F	FGD	61	Christianity	Primary	Trading	<₦30,000	Healthy	11 years
33	Mrs. Can	F	FGD	60	Christianity	Primary	Farming	<₦30,000	Healthy	6 years
34	Mrs. Ano	F	FGD	60	Christianity	No education	Trading	<₦30,000	Healthy	15 years
35	Mrs. Nno	F	FGD	85	Christianity	No education	Unemployed	<₦30,000	Unhealthy	9 years
36	Mrs. Anak	F	FGD	85	Christianity	No education	Unemployed	<₦30,000	Unhealthy	10 years
37	Mrs. Kan	F	FGD	60	Christianity	Secondary	Artisan	<₦30,000	Healthy	2 years
38	Mrs. Obi	F	FGD	62	Christianity	Primary	Farming	<₦30,000	Unhealthy	6 years

*Note*: BSc = Bachelor of Science; F = female; FGD = focus group discussion; FSLC = First School Leaving Certificate; IDI = in-depth interview; M = male; N/I = not indicated; SSCE = Senior Secondary Certificate of Education.

**Table 3.** Sociodemographic Characteristics of IDI and FGD Participants in Igbo-Eze North Local Government Areas

S/N	Pseudonym	Sex	Type of Study	Age	Religion	Education Status	Occupation	Monthly Income	Health Status	Period of Widowhood
1	Mr. Bob	M	IDI	60	Christianity	Primary	Artisan	<₦30,000	Healthy	3years
2	Mr. Wil	M	IDI	79	Christianity	Primary	Artisan	<₦30,000	Healthy	8 years
3	Mr. Ebo	M	IDI	79	Christianity	No education	Farming	<₦30,000	Unhealthy	3 years
4	Mrs. Ono	F	FGD	60	Christianity	No education	Farming	<₦30,000	Healthy	4 years
5	Mrs. Ife	F	FGD	N/I	Christianity	No education	Unemployed	<₦30,000	Unhealthy	20 years
6	Mrs. Nko	F	FGD	60	Christianity	Tertiary	Civil servant	>₦30,000	Unhealthy	11 years
7	Mrs. Udu	F	FGD	N/I	Christianity	No education	Farming	<₦30,000	Healthy	N/I
8	Mrs. Ikwo	F	FGD	N/I	Christianity	No education	Trading	<₦30,000	Unhealthy	24 years
9	Mrs. Ibe	F	FGD	N/I	Christianity	No education	Trading	<₦30,000	Healthy	20 years
10	Mrs. Udi	F	FGD	N/I	Christianity	No education	Trading	<₦30,000	Unhealthy	13 years
11	Mrs. Oyi	F	FGD	N/I	Christianity	No education	Trading	<₦30,000	Unhealthy	12 years
12	Mrs. Oma	F	FGD	60	Christianity	No education	Trading	<₦30,000	Unhealthy	22 years
13	Mrs. Oke	F	FGD	N/I	Christianity	No education	Unemployed	<₦30,000	Unhealthy	17 years
14	Mrs. Cha	F	FGD	60	Christianity	Primary	Trading	<₦30,000	Unhealthy	5 years
15	Mrs. Ukwu	F	FGD	68	Christianity	No education	Trading	<₦30,000	Unhealthy	16 years
16	Mrs. Zed	F	FGD	63	Christianity	No education	Trading	<₦30,000	Healthy	11 years
17	Mrs. Nad	F	FGD	60	Christianity	No education	Trading	<₦30,000	Healthy	11 years
18	Mrs. Oye	F	FGD	60	Christianity	No education	Trading	<₦30,000	Healthy	2 years
19	Mrs. Amad	F	FGD	62	Christianity	No education	Trading	<₦30,000	Unhealthy	15 years
20	Mrs. Ode	F	FGD	60	Christianity	Primary	Artisan	<₦30,000	High	3 years
21	Mrs. Edo	F	FGD	78	Christianity	No education	Unemployed	<₦30,000	Unhealthy	25 years
22	Mrs. Lag	F	FGD	N/I	Christianity	No education	Unemployed	<₦30,000	Unhealthy	17 years
23	Mrs. Jan	F	FGD	85	Christianity	No education	Unemployed	<₦30,000	Unhealthy	35 years
24	Mrs. There	F	FGD	60	Christianity	Primary	Trading	<₦30,000	Healthy	18 years
25	Mrs. Joyo	F	FGD	65	Christianity	Primary	Trading	<₦30,000	Healthy	2 years
26	Mrs. Hel	F	FGD	70	Christianity	No education	Unemployed	<₦30,000	Unhealthy	6 years
27	Mrs. Eke	F	FGD	60	Christianity	No education	Trading	<₦30,000	Unhealthy	1 year
28	Mrs. Osa	F	FGD	65	Christianity	Primary	Trading	<₦30,000	Healthy	7 years
29	Mrs. Ede	F	FGD	65	Christianity	No education	Trading	<₦30,000	Unhealthy	8 years
30	Mrs. Ble	F	FGD	60	Christianity	Primary	Trading	<₦30,000	Healthy	7 years
31	Mrs. Cato	F	FGD	60	Christianity	Secondary	Trading	<₦30,000	Healthy	1 year
32	Mrs. Ezeco	F	FGD	65	Christianity	Primary	Trading	<₦30,000	Healthy	2 years
33	Mrs. Bloso	F	FGD	60	Christianity	Primary	Trading	<₦30,000	Healthy	8 years

*Notes*: F = female; FGD = focus group discussion; IDI = in-depth interview; M = male; N/I = not indicated.

*Source*: Researchers’ fieldwork, 2022.

### Theme 1: Emotional Challenges Experienced by Widowed Older Adults

The study analysis revealed that all the widowed participants experience emotional challenges as a result of the loss of their spouse. The emotional challenges they experience are loneliness, life dissatisfaction, anxiety/uneasiness, sadness/grief, and insomnia.

### Loneliness

Widowed older adults experience loneliness due to the loss of their spouse. Almost all the participants in the FGDs conducted in both LGAs stated that they are challenged in this manner. At Igbo-Eze North LGA, the women noted that they badly feel the demise of their husbands and thereby feel lonely. They said that two are better than one, but when it is only one person that is in the house, the person feels very lonely. What two persons (husband and wife) would have done, only one person is doing it. Similarly, at Nsukka LGA, some of the women revealed that they were neither sick nor suffering from any illness, but they are sick from loneliness. They also revealed that once they were two, but now their husbands have left them, thereby subjecting them to suffer loneliness.

Other female participants in IDIs had similar experiences. A female participant, Mrs. Nky, who had been a widow for 13 years, said, “the first thing a widow experiences is loneliness. I advise that anyone who sees a widow misbehaving should not be judging her, instead, pray for her because if you find yourself in her situation, you may do same thing.” In support of the above assertion, Mrs. Flo, a widow for 22 years, asked:

Why? Am I not a human being? I feel lonely from time to time. The loss of a partner is unforgettable. It is not easy. My children were around me at the initial stage of my widowhood but as time went on, they left home in search of their destinies. So, I am left with my last born who is even not steady at home.

From the analysis, it appears that men also have the same experience brought about by the demise of their wives. Mr. Onu said, “I often feel lonely as a result of my wife’s death, you know, she is my companion.” Another participant, Mr. Uke, said, “I always feel lonely and emotional trauma as a result of the demise of my wife and I am not allowed to attend any social gathering during the period of widowhood.” Also, Prof. Kodo said, “most of the time, I feel lonely. I miss her a lot. Things that a woman would do in the house is left undone, no doubt the vacuum is there, especially when the children were younger.” The opinion of Mr. Bob was reflected in the quote:

Since the demise of my wife, I feel lonely but I do hold myself. I am not into any intimate relationship since she died. I do not want to remarry. I control myself when I have sexual feelings. I may get a female friend later, but for now, I am too busy. I have to face only the stress of raising my children and working to earn money to be taking care of them. When I make much money, I may start thinking of getting a girlfriend.

### Life Dissatisfaction

The study data revealed that some of these older adults said that the death of their spouses made them dissatisfied with life. In the FGD conducted with women at Nsukka LGA, these women noted that they are not satisfied with life. To them, whenever there are events that are meant to make them happy, they tend not to be because they wanted to share the happiness with their partners. Mrs. Ono, whose is 60 and has been a widow for 4 years, said:

The death of our husbands usually makes us to be dissatisfied in life. Whenever our children succeed in life like building houses for us, what your mind will tell you is that, it’s now only you that is entering the house. Where is the person, they built the house for you and him? You will become angry and dissatisfied that he is not there, that person that both of you gave birth to the children is no longer alive to enjoy the things these children are getting or bringing, it always makes us angry.

Similarly, other participants in the IDIs studies revealed that the death of their partners has made them dissatisfied in life. A male participant, Prof. Kodo, chuckled and asked, “of course, when part of you is dead, are you still alive?” Another male participant, Mr. Bob, said, “I was feeling sad and dissatisfied due to the death of my wife. I would have loved to spend my life with her.” In the same vein, Mrs. Flo, who had been a widow for 22 years, exclaimed while she expressed her view:

Aaah! Is it something to talk about? Occasionally I feel like what am I doing in this world. I do feel that, it is better to die, than face the untold suffering that has been my experience especially at the early stage of my widowhood. It is of late that I started wanting to live again because some of my children can now buy bread for their mother when they visit home, life is becoming sweet again but at the same time, I am not enjoying these things because my very half is not there to enjoy the fruits of our labors.

### Anxiety/Uneasiness

Widowed older adults experience anxiety/uneasiness as revealed by the data. A greater number of the participants revealed their constant concern about the family well-being. Others also noted their concern over the financial upkeep of their families without the assistance from their spouses. Mr. Bob, whose wife died 3 years ago, revealed that the task of meeting with the financial responsibility makes him to be anxious. He said, “I always feel anxious on how to be meeting up with finances to be taking care of my children’s needs.” Some of the expressions from the participants were captured in the quotes:

I think a lot. For the past two weeks I have not been sleeping. I am always thinking about family issues, how my children could amount to something. Issues I would have been sharing with my husband, I am now bearing them all alone. I am now having high blood pressure. Because of my situation, I keep thinking, even when I don’t want to be thinking. My children perform comedy for me, surrounding me as if I were to be a child, so that I will stop thinking, but I keep thinking. I don’t like telling my children all that are happening to me, because they too will start thinking. (Mrs. Nky; 60 years; 13 years into widowhood)

Hmmmm! I do feel anxious to the point of questioning God why it seems my own case has become different. People who came to console me when my husband died were assuring me that it was for a while, but it has lasted longer than I imagined. I don’t see the end as something that will come soon for which I worry so much about. (Mrs. Flo; Farmer; 22 years into widowhood)

### Sadness/Grief

The transcript further showed that the participants experience sadness/grief due to the death of their spouses. In the FGDs conducted at Igbo-Eze North and Nsukka LGAs, all the participants noted that they are saddened due to the demise of their spouses. Some of them noted that the inhuman treatment they are subjected to by community members always made them sad. One of the participants narrated how she feels sad whenever she remembers the accusation against her by the community members. She said she was accused of poisoning someone. Despite handing it over to God for justice, she still feels sad whenever the memory overwhelmed her.

Still buttressing the experiences of sadness resulting from the inhuman treatment by the members of the community, another participant revealed that she feels saddened due to the forceful taking away of her husband’s belongings. According to her, such action would not have happened if the husband was alive. Her view is reflected in this quote:

The thing that brings sadness in widowhood is when they are taking away what belongs to your husband and I know that it belongs to two of us and two of us used to go and get things from there, and because he is no more, people are taking it by force. It could be that the person is stealing it, which he could not have done if my husband was still alive for fear of him. These bring sadness in my life. Even those trained by my husband are taking away my husband’s property. (Mrs. Hel; 70 years; 6 years of widowhood)

### Insomnia

The analysis of the transcript revealed that older adults who are widowed also experience insomnia. Some of the participants noted that excessive thinking about the family and how to care for them in the absence of the spouses usually cause them to be awake at night. In the IDIs conducted in Nsukka LGA, a female participant, Mrs. Flo, who had been a widow for 22 years, said, “yes. It is uncountable. I use to go to bed around 10 pm and stay awake until 2 am. No sleep would cross my eyes. Sometimes, this happens for a week at a stretch.” Also, in the FGD conducted with the older adults in Nsukka LGA, Mrs. Ono, who had been a widow for 4 years, said:

Too much thinking causes us sleepless night; thinking of the children, and how to take care of them. This situation worsens our health. Blood pressure has been my major issue for a very long time now, because of too much thinking on how to take care of my children’s school fees, money and food. When you think about this whole thing, you fall sick instantly. If you see my face, you will think I am that too old, but I am a young-old adult only that conditions are not favorable. Sometimes, I get confused about which one to think, whether food or school fees. Because of all these, sleep cannot come my way. Most time, you hiss because the condition is no longer funny. There is no husband to discuss things with or how to go about things in the family, now all burdens is on me to think, provide and when you wake up in the morning, anything you see, you face it all alone for the day.

### Theme 2: Spiritual/Religious Activities Widowed Older Adults Are Participating in

#### Religious activities

Having indicated the emotional challenges participants experience, the transcript further revealed that older widowed participate in religious activities. Almost all the participants strongly indicated their involvement in religious programs or activities. These religious activities include routine weekly church activities like Sunday services, routine prayer meetings, harvest services, and other church activities. In the FGD conducted with widows at Igbo-Eze North LGA, the participants revealed that they participate in Church activities such as Sunday mass and women’s group meetings. They also stated that they attend different harvest services and sanctuary cleaning despite the death of their husbands. Also, at Nsukka LGA, the widows that participated in the FGD revealed that they are involved in cleaning the church every Saturday to ensure that the church premises are tidy. They also engage in routine group prayers in members’ houses on Mondays; attend August meeting, women’s conferences, and mothering Sundays. Finally, they revealed that they contribute money among themselves to solve some of the needs of the churches they attend, especially in emergencies.

Similarly, the IDIs participants indicated that they attend some of the activities in their places of worship. Mrs. Flo, who is a Christian and had been a widow for 22 years, revealed that she regularly attends church services and also other church activities periodically. Her expression was captured in this quote:

I attend church services regularly. I am not found wanting in church programs. I do not attend church programs occasionally. Once it is time for church programs especially prayer meeting, I leave every engagement. I could be in the farm working; I would abandon it half way and go to the church. I do not joke with church activities.

The expression of a male participant, Mr. Wil, whose wife died 8 years ago, was reflected in the quote:

Haw! I go to the Anglican church. Emm … I attend men’s prayer meeting and contribute money … I pray in the morning and pray in the night. I participate in the Church activities. Except that I fall from palm tree last week, I did not go to Church. I am still having the pains.

#### Spiritual activities

The study data revealed that widowed older adults in the study areas engage in spiritual activities. Some of the participants stated their desire to seek or pursue a deeper relationship with God through spiritual activities. These activities include personal prayers, engaging in Bible study, meditation, and personal retreat through fasting. In the FGDs conducted with the widows at Igbo-Eze North LGA, they indicated that they pay attention whenever the word of God is being preached in the church. They said that they are devoted to the word of God, which has been helping them to overcome certain challenges. The participants indicated that their commitment to the word of God has made them to realize that God is even the husband of the widow. Also, their dedication to the word of God through the Bible study has made them to be more dedicated and ensured that spiritual progress is made by the church. They equally stated that they have understood the spiritual significance in giving (money), for the work of God as well as the blessing therein. During the discussion, Mrs. Ukwu, aged 68 and a widow for 16 years, said:

Many things are taught in Bible study that is done during Sunday church service. Many things are learnt from it. If I am to say, I will say that we are learning more from the Bible study than from the sermon. There are ways Bible study handles the word of God that helps us in our families, in the church and our actions in the community. So, the word of God studied during the Bible study helps us a lot. It has made us to understand that we can be among those that will spread the word of God to win souls for Christ. Some of us do it as much as we can and because of this, we are eager to study the word of God independently.

In another FGD conducted with widows in Nsukka LGA, another female participant, Mrs. Obu, who had been a widow for 15 years, said:

We do that. In my family for instance, I gather my children and let them know what the scripture wants us to do. You let them know that God is their father and everything; that they should follow his word, for it is profitable to follow him. There is great gain in following the instructions of God. God knows that if we follow him, it will be well with us. That is why we should follow him.

Similar expressions were made by the participants in the IDIs conducted in other study areas. One of the male participants, Mr. Bob, who is aged 60 and a widower for 3 years, narrated his active involvement in spiritual activities including meditating in the word of God as well as his involvement in personal retreat/fasting. He reflected thus:

For the word of God, I meditate on it daily, and with my family. Sometimes I gather my children to pray. Sometimes we declare fasting for our effective relationship with Him, and to seek for His intervention. We also do that to build our trust in Him.

Another female participant clearly stated that she attends church activities and engages in personal meditation. She quoted thus:

As a full member of the [name of church hidden], I started attending prayer meetings in the church, doing personal prayers, night vigils, attending every of the church’s programs, I read and meditate on the Bible every day, sweep the church, and participate in all the church activities. (Mrs. Nky; Christian; 13 years into widowhood)

### Theme 3: Extent to Which Spiritual and Religious Activities Have Aided Widowed Older Adults

#### Religious activities

All the female participants in the FGDs conducted in the areas revealed that after attending these religious activities, they are encouraged and comforted. During the discussion at Igbo-Eze North, the women noted that whenever they pray, get involved in church activities, they are comforted, and the Holy Spirit comes into their soul to comfort them. At Nsukka, a widow, Mrs. Obi, who is a Christian and had been a widow for 6 years, said this during the discussion:

When I go to church, and hear the words of God, it is as if I am the one the words are meant for, to address my needs. Some of the preaching moves me to be shedding tears. As I am going home, I understand it to be tears of joy, I will be happy within myself.

Out of excitement, one of the female participants in the FGD conducted at Igbo-Eze North LGA, Mrs. Zed, who is aged 63 and had been widowed for 11 years, shouted:

Thank you, Jesus Christ! Hmmm … emm … where do I start from self …? These prayers and church services we attend have really helped to lift our burdens. Sometimes, when I am on the way to the Church, for services or programs, I used to be moody but, on my way back, I would become a different person, lively, excited, with hope rekindled. That is much this Church thing has helped us.

Going further, the participants in the IDIs conducted in the areas also show that participating in religious activities has aided them to cope with the challenges of widowhood. A male participant noted how these religious activities have helped him. He reflected:

Attending church services, praying with my children at night and attending morning mass regularly helps me to buffer the challenges of widowhood and other life challenges. Participating in church activities helps me to be spiritually strong. Anything that is bothering me, after I attend morning mass, go to chapel and pray, after praying God will just open way to solve that problem. (Mr. Bob, 3 years in widowhood)

In the same vein, a female participant said:

Church activities help so much, and stopped a lot in me, I am now having joy and happiness. Loneliness has gone away. If I have problems, I will go and share it with the pastor and the pastor is there for me. The pastor tackles my problems immediately. Being that the church has small number of members, we understand ourselves. We do not permit even one of our members to go away; it is now as if I have gotten another family. (Mrs. Nky; aged 60 and 13 years a widow)

Another female participant in the IDIs, Mrs. Ros, who is a healthy trader and had been a widow for 11 years, indicated how participating in religious activities has enabled her to be busy and to be connected socially. She said:

My engagements with these activities [religious] have helped me a lot to keep busy. God has shown me so much mercy. I see my engagement in these religious activities as a way of relating with God to keep protecting my children and me. Being busy has also helped me to think less. Reaching out socially has helped me tackle some problems emanating from widowhood as lessons got from group discussion have been a great source of finding solution to my own personal problem.

#### Spiritual activities

The study data revealed that the participants in the IDIs conducted in the areas show that participating in spiritual activities has aided these individuals to cope with the challenges of widowhood. The reading and meditating on the word of God have really helped them. A female participant recalled how she found succor and comfort from the book of Job. She said; “mgbe di m nwụrụ, echetara m akụkọ maka Job. O wee n’enye m nkasiobi” [when I lost my husband, I remembered the story of Job. It gives me consolation]. Another remarked, “ewezuga okwu chineke na ndụ m, m gabụgo onye nwụrụ anwụ” [if not for the word of God in my life, I would have been dead by now]. In support of the above statement, Mrs. Flo, who had been a widow for 22 years, said:

I do meditate. I use psalms a lot in my meditation. I also meditate on the story of Job. The story of Job was a big consolation to me when I compared what befell me with that of Job. I usually ask myself; if Job was able to pull through his experience, I know one day mine would be story.

Mr. Bob, who is aged 60 and had been a widower for 3 years narrated his active involvement in spiritual activities including meditating in the word of God as well as his involvement in personal retreat/fasting. He reflected thus:

If the situation is worse in the area of money; let’s take for instance, as Christmas is approaching and we are unsure of what resources for the celebration, then I will tell my children that we will schedule fasting, and prayer concerning it. Call on God who is in heaven who is our trust, who said if we trust Him, we will not be shamed. So, we pray and call on Him and God makes name for himself for he answers prayers.

## Discussion

The study investigated how involving in spiritual/religious activities has helped widowed older adults to buffer their emotional challenges. The study was conducted in Enugu state, southeast Nigeria. The findings of the study revealed that these individuals feel lonely because their partners who kept them company are no more. They are dissatisfied because they no longer share the joy of beautiful things around them. They also feel unhappy due to the maltreatments from some of the members of community, and these conditions made them not sleep consequent upon excessive thinking. The study findings can be explained by the import of COR, which revealed that when resources are lost, it leads to the experience of emotional or psychological pain (Hobfoll, 1989). These findings are in tandem with some studies that revealed that German and Korean widowed older adults experience anxiety, grief, depression, emotional loneliness, and social loneliness (Förster et al., 2019; Kim et al., 2018). In China, spousal deaths occasioned some emotional challenges like depression and anxiety (Bi et al., 2022; Tian & Chen, 2022). Similar findings have been obtained in other studies across various climes (Battle, 2013; Brouwer et al., 2022; Domingue et al., 2021; Singham et al., 2021; Szabó et al., 2020).

The study findings revealed that widowed older adults participate in religious and spiritual activities. Some of these older adults attend more church activities, others hold more to personal and family spiritual activities. These individuals participate in activities like vigils, revival programs, different prayer meetings, harvest services, and sanctuary cleaning. They also engage in personal spiritual activities like Bible study and meditation, family devotion in the morning and at night. In agreement, COR theory revealed that even when the resources are lost, the individuals can also regain more resources (Hobfoll, 1989). The resources regained usually act as a buffer at the point of losing resources (Farkash et al., 2022). Whenever individuals lose resources, they make every effort to regain them to enable them to secure their emotional strength for achieving desired goals (Wells et al., 1999). Similarly, Carr (2020) revealed that one of the coping strategies used by widows is turning to God through prayers, Bible study, and meditation. In the same vein, the study by Ben-Nwankwo (2014) revealed that widowed older adults are deeply involved in spiritual activities like prayer either at home or in the sanctuaries.

Support received from participating in these activities by widowed older adults in coping with emotional challenges was revealed by the study. By engaging in religious/spiritual activities, widowed older adults were found to buffer some of the emotional challenges. Explaining the finding of the study is the COR theory, which posits that individuals can regain more resources even when the resources they struggle to retain, protect, and build are threatened through loss (Hobfoll, 1989). The loss of the resources usually leads to emotional pain; thus, the individuals usually turn their attention to the best way to secure their emotional well-being for achieving desired goals (Wells et al., 1999). The finding corroborates views of earlier studies, which revealed that widowed older adults buffer some challenges associated with widowhood by putting their faith in God through their involvement in religious and spiritual activities (Abiola & Ndisik, 2022). Spiritual and religious activities have enabled widowed older adults to reduce the occurrence of anger and sorrow resulting from the deaths of their spouses (Carr, 2020). Spiritual activities are essential for the well-being of older adults, providing them with necessary support for life satisfaction and sense of purpose (Pilger et al., 2017).

All the participants in the study noted that loneliness is felt consequent upon spousal loss. In the study areas, emotional bonding is usually strong between a couple, the death of one resulting in permanent separation, which is difficult to heal. As a result, widows generally look up to God to take the place of their husbands. They agreed that God has become their husband. Participants are convinced that their belief in God and their conversation with God in prayers helped them assuage the aching void left by the loss of one’s spouse. Thus, they are encouraged to participate in spiritual activities. Church leaders should make every effort to organize activities that will address the emotional needs of widowed older adults. Ebimgbo et al. (2018) found that the periodic teaching programs by the church have been helpful in developing the mind of older adults.

The study also found that reading, studying, and meditating on God’s word were helpful to widowed older adults in dealing with emotional challenges. They read and meditate upon the word of God. Their interest in attending Bible study in churches and other fellowship meetings increased due to the encouragement they got from the word of God. Similarly, the study by Neimeyer (2005) revealed that participation of widowed older adults in religious and spiritual activities gives them hope and reasons to stay put and cope with the spousal loss. Their belief in God through spiritual activities provides them with needed attributes to overcome the experiences of anger and injustice, and other emotional consequences (Davis et al., 2000). The COR theory by Hobfoll (1989) revealed that the loss of resources by the older adults could lead to the effort to regain the resources to maintain stable life course. In most cases, for widowed older adults, participating in religion and spiritual activities enabled them to buffer several challenges associated with widowhood experiences.

As with many other studies conducted across the world, our study is not devoid of limitations. First, we recognized that the study was able to highlight the views of widowed older adults who seemed to belong to almost one religious sect (Christianity) thereby negating the views of other religious sects. We presumed that this was as a result that the study was conducted in one geopolitical zone of the nation. We believe that this may have affected the generalization of the data. We therefore suggest further studies take into consideration the views of other widowed older adults with other religious backgrounds and in other geopolitical zones of the nation. Second, the study only took cognizance of the emotional well-being of widowed older adults without considering other roles expected of faith-based organizations in addressing the financial, material, health, and other needs of widowed older adults. We also suggest further studies that will address these vital issues.

## Conclusion

There is an apparent increase in the population of individuals who are 60 years and older in Nigeria. Noticeable, too, is the surge in the population of widowed older adults. The life of widowed individuals could be challenging and traumatic, especially in the later days of life. This study reveals that among other things, widowed adults experience emotional trauma like loneliness, life dissatisfaction, anxiety/uneasiness, sadness/grief, and insomnia. In order to douse the above ugly experiences, almost all the participants strongly indicate their attachment to spiritual/religious activities. There is a strong indication that the attachment of widowed older adults to spiritual/religious activities brings great encouragement and succor to them. Thus, religious groups through their leadership should create a forum for the widowed to help take care of them through special programs that address their emotional, social, information, financial, and material needs. Religious organizations should also collaborate with other stakeholders to develop an early warning system to protect the widows’ rights against some obnoxious treatments usually meted out on them.
